# The cytotoxic, genotoxic and mitotoxic effects of *Atractylis gummifera* extract in vitro

**DOI:** 10.4314/ahs.v24i1.35

**Published:** 2024-03

**Authors:** Awatif Boumaza, Ali Ergüç, Hilmi Orhan

**Affiliations:** 1 Department of Biology, Faculty of Nature and Life Sciences and Earth and Universe Sciences, Université 8 Mai 1945, Guelma, Algeria; 2 Department of Pharmaceutical Toxicology, Faculty of Pharmacy, Ege University, 35040 Bornova-İzmir, Turkey; 3 Department of Pharmaceutical Toxicology, Faculty of Pharmacy, Katip Celebi University, Balatçık Campus 35620 Çiğli İzmir, Turkey

**Keywords:** *Atractylis gummifera*, *Allium cepa* test, mitochondrial permeability transition pore, genotoxicity, cytotoxicity, chromosomal aberration, mitotic index

## Abstract

**Background:**

The Mediterranean thistle *Atractylis gummifera* L. (Asteraceae; AG) has diterpenoid glucosides; atractyloside and carboxyatractyloside that interact with mitochondrial protein adenine nucleotide translocator (ANT) and resulted in ATP inhibition. Despite its well-known toxicity, acute poisonings still occur with this plant. Although most symptoms are attributed to ANT and diterpenoids interaction, in-depth investigation of the effects of AG extract on various cellular processes has not been performed.

**Objective/method:**

We tested *in vitro* induction of mitochondrial permeability transition pore (MPTP) opening in bovine liver mitochondria and evaluated its cytotoxicity and genotoxicity using *Allium cepa* test. Cell division, mitotic index (MI) and total chromosomal and mitotic aberrations (TAs), that all seem potentially affected by ATP shortage, were studied in root cells of *Allium cepa* exposed to *Atractylis gummifera* extract.

**Results:**

With the two different doses of two purified AG fractions, stronger induction of MPTP was observed compared to the induction with the standard pure atracyloside. Aqueous AG extract exerted inhibition root growth in *A. cepa* at 6 different doses. The TAs was increased in a dose-dependent manner too, while mitotic index was decreased at the same doses. Evaluation of mitotic phases revealed mitodepressive effect of AG on *A. cepa* roots.

**Conclusion:**

this work highlights cellular and mitochondrial adverse effects of *Atractylis gummifera* extracts. A purified fraction that likely corresponds to ATR derivatives induces MPTP opening leading to swelling of mitochondria and its dysfunction. *Allium cepa* test provides the evidence for *A. gummifera* genotoxicity and cytotoxicity.

## Introduction

The Mediterranean thistle *Atractylis gummifera* L. (Asteraceae; AG) has long been known as toxic both to humans and animals. Serious health complications, acute and fatal intoxications are recorded in North Africa (Algeria, Morocco, and Tunisia) where this plant is known as glue thistle and used in aesthetic as a natural scrub, to lighten the skin and eliminate dark spots and skin pigmentation treatment. It is used in alternative veterinary medicine against parasites and in traditional medicine to treat intestinal parasites, ulcers, snakebite poisonings, hydrops and many other symptoms 1. The toxicity of AG seems to be attributed to the presence of atractyloside (ATR) and carboxyatractyloside (CATR); that are toxic diterpenoid glucosides interacting with protein adenine nucleotide translocator in mitochondria (ANT) and resulted in ATP inhibition, thereby blocking oxidative phosphorylation, which inhibits ATP synthesis and causing the failure of gluconeogenesis and cell death 2. It has been reported that ATR inhibits energy processes in both kidney and liver tissues and exhibits cytotoxicity *in vitro* 3. ATR is also thought to lead to apoptosis via the mitochondrial permeability transition pore activation (MPTP), which leads to the liberation of cytochrome c and caspase-activating proteases. Cellular damage is caused by reduced ATP/ADP ratios and increased mitochondrial production of reactive oxygen species (ROS) that can damage cells macromolecules like proteins, lipids and DNA. ROS are well recognized as genotoxic and mediators of DNA damage. They have been reported to directly induce DNA damage through oxidizing nucleoside bases (e.g. formation of 8-oxoguanine) and mitochondrial DNA lesions, strand breaks and degradation of mitochondrial DNA. They also lead to replication stress ultimately results in genomic instability 4. While these data are supporting the adverse effects of AG, a recent *in vitro* and *in vivo* study of Khadija Bouabid *et al* 4 showed that aqueous and organic extracts of AG protect against lipid peroxidation and induce the enzymatic antioxidant SOD. They concluded that their results support the use of aqueous extract in traditional medicine, but further toxicity studies are required to determine the safe therapeutic doses 5. Lu Qi *et al.* showed that ATR may potentially target cancer-associated fibroblasts and inhibit the metastasis of colon cancer 6. Recent findings regarding ANTs and their important mechanisms in cancer, with a focus on the therapeutic potential of targeting ANTs for cancer therapy were reviewed by Lin Zhao *et al* 7. They reported a possible therapeutic effect of ATR and CATR via ANT in cancer treatment. Although most symptoms are attributed to ANT and diterpenoids interaction, in-depth investigation of the effects of AG extract on various cellular processes has not been performed. The aim of the present study was to test *in vitro* effect of AG extract on MPTP opening in isolated bovine liver mitochondria, and to evaluate its cytotoxicity (cell division, mitotic index; MI) and genotoxicity (total chromosomal and mitotic aberrations; TA) in root cells of *Allium cepa*, that all seem may potentially be affected by ATP shortage.

## Materials and methods

### Chemicals

Sucrose, mannitol, acetonitrile, methanol, sulfuric acid, chloroform and formic acid were obtained from Merck (Darmstadt, Germany). Bradford reagent was purchased from BioRad (St. Louis, USA). Bovine serum albumin (BSA) was purchased from Santa Cruz (Dallas, USA). All other chemicals were purchased from Sigma Aldrich (St. Louis, MO, USA).

### Plant collection and extracts preparation

AG roots were collected during March from the region of Hammam Debagh (Beni-Addi) in Guelma City, Algeria. The samples were well washed and rinsed with distilled water, then, they were dried in an oven at 40°C for one week. The dried roots were powdered and stored in a tightly sealed bottle at 4°C until use. The methanol extract was prepared by macerating AG roots powder in 70% methanol for three days. The filtrate of AG roots methanol extract was concentrated with rotary evaporator at 40°C, then lyophilized and conserved in a well-sealed container at 4°C until use. AG water extract was freshly prepared by pouring boiling synthetic water on weighted root powder then kept for 30 minutes. To determine the final concentration, 1mL of the infusion was dried at 40°C temperature until constant weight. The final concentration was then measured.

### Purification of AG extract

Dried methanolic extract of AG was dissolved (3 mg/mL) in methanol. Solution was centrifuged at 13000 x g and 25°C for 10 min to remove possible insoluble particles and obtain a clear supernatant. Supernatant was transferred into new vial and analyzed by prep-HPLC at 3 mL/min flow rate and at 200 nm wavelength. Prep-HPLC separation was carried out with an XBridge Prep Shield RP18 (10x10mm, 5µm) column using a Thermo Scientific prep-HPLC system equipped with an Ultimate 3000 autosampler, pump, column oven, and UV-detector. 100 µL of supernatant were analyzed using a gradient elution described below over 30 min at 25°C. Mobile phase A is consisted of water including 0.05% formic acid and mobile phase B is consisted of 100% acetonitrile. Fractions of AG extract was detected at 200 nm wavelength. Composition of gradient mobile phase used for purification of *Atractylis gummifera* extract was as follows:

**Table:** 

Time (min)	Mobile Phase A (%)	Mobile Phase B (%)
0	90	10
20	75	25
24	60	40
25	90	10
30	90	10

Fractions of AG extract were applied to thin layer chromatography in order to check the purity by using chloroform: methanol: water (61:32:7) at 365 nm wavelength. Two purified fractions and total methanolic extract of AG were applied to silica gel on a plate to compare impurity. The plate was exposed to 10% sulfuric acid and heat for visualization.

### Isolation of mitochondria from liver tissue

Mitochondrial fraction was isolated from fresh liver tissue of bovine by differential centrifugation, as described elsewhere 8,9 with minor modifications. 1 g of liver tissue was placed in Petri dish and minced with a scalpel on ice. Tissue was transferred into Pyrex tubes and homogenized with a Teflon pestle using 10 strokes in 10 mL ice-cold sucrose-mannitol buffer (70 mM sucrose, 220 mM mannitol, 20 mM HEPES, 2 mM EDTA, 0,5 mg/mL BSA, pH: 7,5). After homogenization, liver lysate was suspended in 40 mL of sucrose-mannitol buffer. Homogenate was transferred into Falcon tubes and was centrifuged at 600xg (4°C) for 10 min to remove nuclei and cell debris. Supernatant was transferred into new tubes and was further centrifuged at 8000 xg (4°C) for 15 min to sediment crude mitochondria. Crude mitochondria pellet was suspended in 10 mL sucrose-mannitol buffer and centrifuged at 8000 xg (4°C) for 15 min to wash. This process was performed two times. After washing steps, sucrose gradient was performed to purify crude mitochondria. To do this, the pellet was suspended in 1 mL 0.8 M sucrose and layered over 6 mL of 0.8 M sucrose. The fractions were centrifuged at 14000 xg (4°C) for 30 min. to purify mitochondria. After centrifugation, pellet was washed twice in sucrose-mannitol buffer at 8000 xg (4°C) for 15 min. Pure mitochondria were stored at 4°C until use on the same day.

### Protein determination

Protein concentration was determined according to the Bradford method with some modifications using BSA as a standard 10.

### Assessment of mitochondrial permeability transition pore (MPTP) induction

Mitochondrial swelling as the indicator of MPTP opening was measured from the decrease in absorbance at 540 nm according to previously published method 9. Bovine liver mitochondria (1 mg/mL) were incubated with AG1 and AG2 fractions (100 and 200 µM), as well as pure ATR (100 µM) in the incubation buffer (125 mM sucrose, 50 mM KCl, 5 mM HEPES, 2 mM KH_2_PO_4_, 1 mM MgCl_2_, pH 7.2) for 10 min. Incubation of AG fractions and ATR were performed either with or without adding CaCl_2_ (250 mM final) as MPTP inducer. The absorbance of the samples was monitored for 10 min. with 1 min intervals. Reduction of absorbance demonstrates the increase in mitochondrial swelling. 1% DMSO was used as solvent control.

### Cytotoxicity and genotoxicity of AG

#### Test organism and growth conditions

Bulbs (2.5 - 3.5 cm) of common onion (*A. cepa* L. 2n = 16), untreated with pesticides were used for the study. The *Allium* test was carried out according to Fiskesjö 11 and Rank 12. The brownish outer scales were removed leaving the ring of root primordia intact. The experiment was performed at room temperature (at 20 ± 4°C) and far from direct sun light

### Cytotoxicity and root growth inhibition test

Cytotoxicity assay is performed as 96 h semi-static exposure test as described elsewhere 12, 13. Six concentrations of AG aqueous extract were used. First, the bulbs were put on test tubes containing synthetic water, which was changed every 24 h. Series of five bulbs with roots of 15–20 mm in length are used for each concentration and control group. The test samples of AG (2, 4, 6, 8, 10 and 12 mg/ml) were freshly prepared in synthetic water (pH = 6.5). The test solutions were daily replaced by fresh solutions. After 96 h exposure, the length of the whole root bundle was measured as described by Fiskesjö 11. EC50 value is estimated and other macroscopic signs of toxicity were also examined. After 96 h, all roots exposed to AG are allowed to recover in synthetic water for 24 h.

### Genotoxicity assay

Genotoxicity assay is performed with three sample concentrations based on EC50 value (1/2 EC50, EC50 and 2EC50). Synthetic water was used as negative control. Five onions are exposed to each concentration under the same laboratory conditions described above. For the first 24 h, the onions are grown in synthetic water, where after they are exposed to AG aqueous extract for 48 h. The test solutions are changed after 24 h. At the end, onions were prepared for microscopy. The root tips were immediately placed in a chilled Carnoy's fixative for 24 h at 4°C, and then they are conserved in 70% ethanol at 4°C until use. For the microscopic observation, five slides were prepared for each test group and randomly coded and scored blindly at 60 x magnification. The root tips were hydrolysed during 8 min in 1N HCl at 60°C and stained by the Feulgen, then the apical 2 mm were squashed in a drop of 45% acetic acid on slides. Cover slips were sealed to the slides using clear fingernail polish 13-15 and a minimum of 500 cells were scored per tested concentration.

### Mitotic index, mitotic phase and total chromosome and mitotic aberrations analyses

For Mitotic index (MI), it can be calculated through the number of cells observed in Prophase (P) + Metaphase (M) + Anaphase (A) + Telophase (T) and can be recorded as a percentage using the formula: MI = [(P+M+A+T)/ Total number of cells] x 100. 16

For mitotic phase (MP), and Total chromosomal and mitotic aberrations (TAs) (bridges, breaks, stickiness, C-mitosis, laggards, and other disturbances), the different stages of mitosis were counted per concentration and were calculated as the proportion of cells in each division phase, and abnormal cells per total examined cells, respectively and expressed as a percentage as described by Kwankua *et al.* 17.

### Statistical analysis

For MPTP assay, data are expressed as the mean ± SEM. Statistically significant differences between groups were analyzed by using one-way analysis of variance (ANOVA) and comparisons were made with Mann-Whitney U test with a confidence level of 95% using GraphPad Prism Software, version 5 (San Diego, CA, USA) for Windows. For cytotoxicity and genotoxicity assay, ANOVA with Newman-Keuls test for multiple comparison was performed using XLSTAT for windows. In all cases, significance was accepted when p-value was lower than 0.05.

## Results

### Purification of *A. gummifera* methanolic extract by preparative HPLC

**Figure 1:**
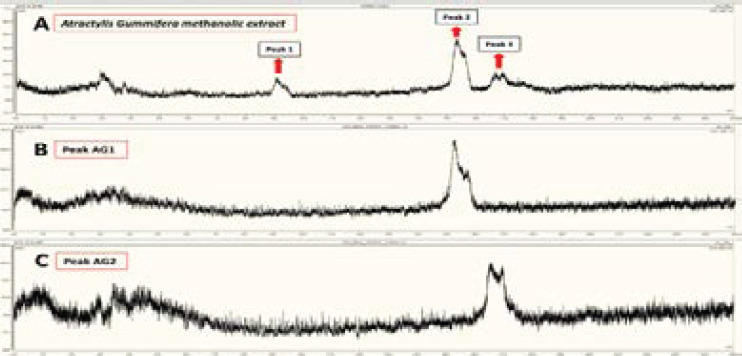
Representative preparative HPLC-UV chromatograms of methanolic extract of *Atractylis gummifera* at 200 nm. A, total chromatogram of methanolic extract before purification; B, chromatogram of peak 2 (AG1) after purification; C, chromatogram of peak 3 (AG2) after purification. Other details were explained in the materials and methods section

Figure 1 represents chromatograms of total and purified AG methanolic extract by preparative HPLC-UV at 200 nm. Basically, 3 peaks were detected (Figure 1A). We collected all three peaks for further purification however, peak 1 did not yield a solid material upon freeze drying. Peaks 2 and 3 were named as AG1 and AG2, respectively (Figure 1B and C).

### Checking impurity of *A. gummifera* fractions by TLC

**Figure 2:**
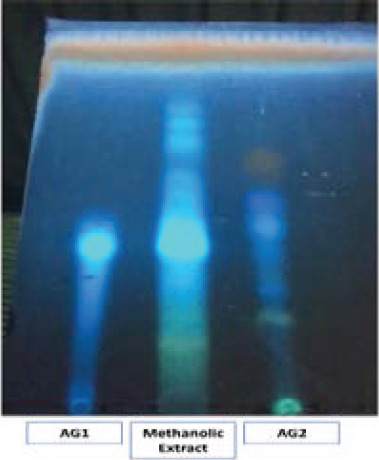
Control of impurities of AG1 and AG2 using silica gel by TLC at 365 nm. Methanolic extract of AG was also loaded onto the plate to compare. Silica gel was exposed to %10 sulphuric acid and heat for visualization. Other details were explained in material methods

Impurity analyses of AG1 and AG2 fractions were performed by TLC at 365 nm (Figure 2). Methanolic extract of AG was also added to silica gel to observe impurities. Our results indicated that AG1 and AG2 were highly pure compared to the total AG extract (Figure 2).

### Assessment of mitochondrial permeability transition pore (MPTP) induction

**Figure 3:**
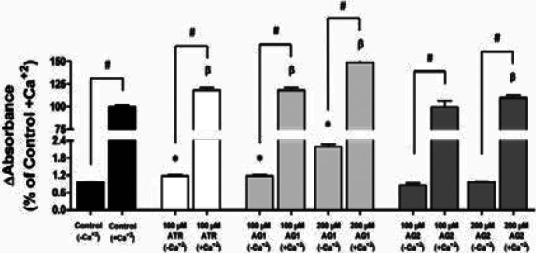
Effect of ATR (100 µM), AG1, AG2 (100 µM and 200 µM) on the swelling of mitochondria (1 mg/mL) in the presence and absence of the inducing agent (250 mM Ca^+2^). Each bar represents average of three independent experiments and values are expressed as mean ± standard error of the per cent of %1 DMSO+Ca^+2^. *, p<0.05 compared to %1 DMSO-Ca^+2^; β, p<0.05 compared to %1 DMSO+Ca^+2^; #, p<0.05 compared to the indicated bar

Effects on MPTP formation of ATR (100 µM), of AG1 and AG2 (100 µM and 200 µM) were investigated in the presence and absence of the inducing factor (250 mM Ca^+2^). ATR at 100 µM and AG1 at both concentrations significantly increased formation of MPTP both in the absence and presence of inducing factor, while AG2 at both concentrations caused a significant increase in MPTP only in the presence of inducing factor (Figure 3).

### Cytotoxicity and genotoxicity of AG

#### Cytotoxicity and root growth inhibition test

The effect of AG aqueous extract on *A. cepa* roots growth is shown in Table 1. The results showed significant dose-dependent inhibition of *A. cepa* roots growth with all tested concentrations of AG when compared to the control (p < 0.05). The estimated effective concentration EC50 that inhibits 50% of root growth is 5.68 mg/ml. Brownish roots with dark tips are observed with concentrations from 6 to 12 mg/ml. Swollen and friable roots are observed with concentrations 10 and 12 mg/ml where some roots are even broken. While concentrations 2, 4 and 6 mg/ml showed moderate roots growth recovery (2 mm) after 24h, no roots growth recovery was observed with the other concentrations of AG. This reveals serious toxicity to *A. cepa*.

**Table 1:** Effect of *A. gummifera* on roots growth of *Allium cepa*

Concentrations	Means of roots growth	Roots growth inhibition
(mg/ml)	(%) + SD*	(%) + SD*
Negative control - 96h	100.00 a	0.00 a
2	84.07 ± 11.86 b	15.92 ± 10.61b
4	68.57 ± 18.22 c	31.42 ± 16.30 c
6	49.09 ± 9.93 d	50.91 ± 8.88 d
8	29.02 ± 3.53 e	70.98 ± 3.17 e
10	12.89 ± 8.03 f	87.11 ± 7.18 f
12	11.64 ± 6.31 f	88.36 ± 5.64 f

SD, Standard Deviation.

* Means with the same letters do not significantly differ at 0.05 level. Comparison is done along the column between different concentrations, and negative control vs different concentrations

### Genotoxicity assay

For genotoxicity study, three concentrations are chosen based on EC50 value: 3, 6 and 12 mg/ml. Table 2 summarizes the effect of *A. gummifera* on mitotic index and mitotic phases of *Allium cepa* root meristemic cells. The percentage of mitotic index (MI%) significantly decreased with the three tested concentrations when compared to the control group and in a dose-dependent manner. Interphase percentage increases when MI% decreases and the accumulation of cells in interphase is significantly different in the three tested concentrations in comparison with the control group. Concerning dividing cells, a significant increase of prophase phase percentage was observed with 6 and 12 mg/ml concentrations in comparison to the control. No significant increase of prophase phase percentage was observed with 3 mg/ml.

**Table 2:** The effect of *A. gummifera* on mitotic index and mitotic phases of *Allium cepa* root meristemic cells

Concentrations	Dividing cells	Means of MI	Interphase	Mitotic phases (%) ± SD*			
(mg/ml)	(%) ± SD	(%) ± SD		Prophase	Metaphase	Anaphase	Telophase
Negative control-48h	87.74 ± 3.19 a	8.77 ± 0.32 a	12.26 ± 3.19 a	29.5 ± 1.73 a	24.65 ± 0.92 a	16.76 ± 2.86 a	16.82 ± 2.80 a
3	63.53 ± 6.63 b	6.35 ± 0.66 b	36.46 ± 6.64 b	30.77 ± 4.74 a	19.07 ± 3.18 b	7.75 ± 1.76 b	6.28 ± 3.12 b
6	43.71 ± 2.48 c	4.37 ± 0.24 c	56.28 ± 2.48 c	25.60 ± 1.15 b	14.16 ± 2.87 c	2.81 ± 1.68 c	1.12 ± 0.42 c
12	8.67 ± 1.68 d	0.87 ± 0.16 d	91.33 ± 1.68 d	6.50 ± 0.84 c	1.27 ± 0.73 d	0.70 ± 0.91 c	0.19 ± 0.18 c

SD, Standard Deviation.

* Means with the same letters do not significantly differ at 0.05 level. Comparison is done along the column between different concentrations, and negative control vs different concentrations

### Chromosome and mitotic aberrations

The effect of AG water extract on chromosome and mitotic aberrations in *A. cepa* is presented in Table 3. Dose-dependent and significant increase of the percentage of TAs was observed in the tested concentrations comparing to negative control.

**Table 3:** Chromosome and mitotic aberrations in root meristematic cells of *Allium cepa* after the treatment with different concentrations of *A. gummifera* for 48 h

Concentrations	Aberrations (%) (Mean ± SD) *								TA % ± SD
(mg/ml)	Breaks	Bridges	C-mitosis	Lagards	Vagrant	Stickiness	MN	Others	
Negative control-48h	0.28 ± 0.27	0.08 ± 0.11	0.28 ± 0.30	0 ± 00	0.04 ± 0.09	0.24 ± 0.26	0.04 ± 0.09	0.6 ± 0.2	1.56 ± 0.52a
3	0.72 ± 0.63	0.84 ± 0.64	2.92 ± 1.20	2.44 ± 0.57	0.48 ± 0.39	3.56 ± 0.82	1.32 ± 0.70	4.32 ± 2.07	16.6 ± 1.64b
6	2.72 ± 1.32	1.2 ± 0.96	5.92 ± 2.16	3.44 ± 1.77	0.68 ± 0.99	9.08 ± 3.59	3.08 ± 1.60	10.92 ± 4.59	37.04 ± 3.02c
12	1.00 ± 0.46	2.52 ± 0.76	6.6 ± 1.6	3.00 ± 1.44	1.44 ± 0.76	9.52 ± 1.43	9.2 ± 2.15	30.2 ± 4.71	63.48 ± 4.73d

SD, Standard Deviation.

* Means with the same letters do not significantly differ at 0.05 level. Comparison for TA% is done along the column between different concentrations, and negative control vs different concentrations

**Figure 4:**
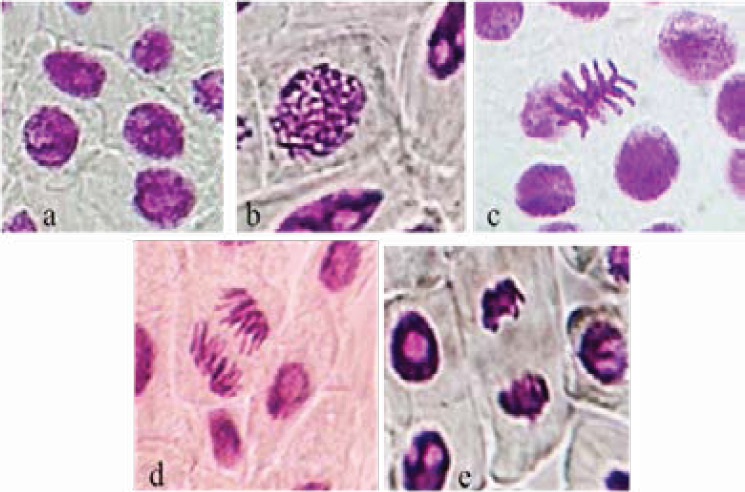
Different normal mitotic phases observed at 60 x magnification in *Allium cepa*: (a) Interphase, (b) Prophase, (c) Metaphase, (d) Anaphase, (e) Telophase

Figure 4 demonstrates normal stages of mitotic division observed at 60 x magnification.

**Figure 5:**
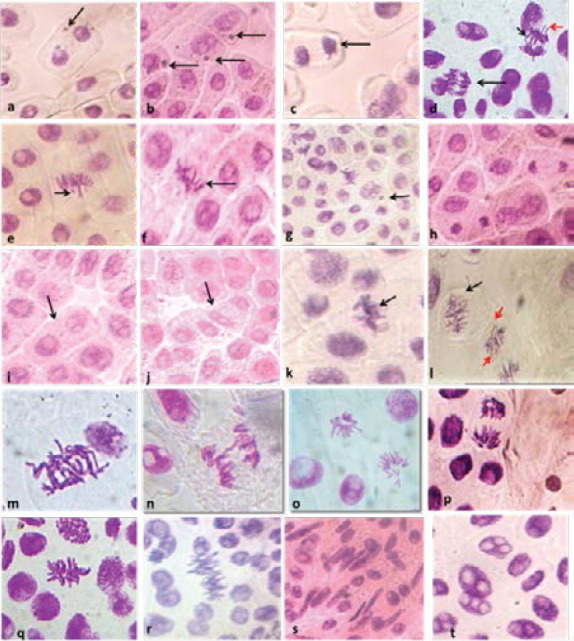
Different chromosome and mitotic aberrations observed at 60 x magnification in *A. cepa* root cell tips exposed to different concentrations of AG aqueous extract (a) binucleated cell with micronuclei, (b) micronuclei in interphase cells, (c) sticky telophase with laggard chromosome (red arrow), (d) multibridge in anaphase + Vagrant chromosome (red arrow), (e) breaks, (f) vagrant chromosome, (g) micronucleus in anaphase (black arrow), (h) Sticky telophase, (i) binucleated cell, (j) Polynucleated cell (4 nuclei), (k) Sticky metaphase, (l) C-metaphase (black arrow) + Anaphase with laggard and vagrant chromosomes (red arrows), (m) anaphase bridges with vagrant chromosome, (n) anaphase bridge, (o and p) disturbed telophase with vagrant chromosomes, (q) C-metaphase, (r) duplicated chromosomes number in metaphase, (s) buds and abnormal nuclei shapes, (t) fragmented nucleoli

Different types of chromosome and mitotic aberrations were observed at 60 x magnification (Figure 5). The results reveal the presence of breaks, bridges, c-mitosis, laggards, vagrant chromosomes, sticky telophase; micronuclei; abnormal nuclei, fragmented nucleolus etc. The predominant aberration types observed with the three concentrations of AG are, in descending order, stickiness, C-mitosis, laggards and micronuclei.

## Discussion

*Atractylis gummifera* is a plant with medicinal and aesthetic importance. Despite its wide use and benefits in alternative medicine, there are many reports on its toxicity and acute/fatal poisonings in humans. However, in-depth investigation of the effects of AG extract on various cellular processes has not been performed and more data are required on its toxic effects especially on the genetic component which has never been studied before. In the present study, toxicity of AG is evaluated through different endpoints. We therefore tested in vitro induction of mitochondrial permeability transition pore (MPTP) opening in isolated bovine liver mitochondria and evaluated its cytotoxicity and genotoxicity in *Allium cepa*.

### Mitochondrial permeability transition pore (MPTP) opening

Permeability transition is a pathological status resulted of the opening of a voltage-dependent and high conductance channel 18. MPTP has been repeatedly linked to mitochondrial dysfunction. Prolonged opening of MPTP results in mitochondrial depolarization, oxidative phosphorylation uncoupling, and reactive oxygen species (ROS) production 19. In the present work, two purified fractions (AG1 and AG2) from AG methanolic extract were used to assess their effect on MPTP opening in mitochondria. Studies on the chemical composition of *A. gummifera* are rare. According to Mejdoub *et al.* 20, only a few saponins, flavonoids, triterpenoids and phenolic compounds were identified. Some studies showed that methanolic extract of *A. gummifera* roots contains polyphenols: flavonoids and tannins 21, 22 and terpenoides (ATR and CATR) 23, 24.

The results showed significant and dose dependent MPTP opening by AG1 with and without Ca^++^. At 100 µM, MPTP inducing effect of AG1 in the absence of Ca^++^ was similar to the pure ATR (used as positive control) at the same concentration. AG2 fraction had no effect on MPTP opening in the absence of Ca^++^. Daniele *et al* 25 isolated both ATR and CATR from the rhizomes of *Atractylis gummifera*. However, CATR is present only in fresh plants. During ageing or desiccation, CATR is decarboxylated to ATR 25. As methanolic extract was prepared from dried powder of AG rhizomes and AG1 produce MPTP opening similar to the pure standard ATR used as positive control, the purified AG1 fraction may be ATR. It is known that Ca^2+^ is an activator of MPTP^26^, AG1 seems to be a potent MPTP opening inducer that produces mitochondrial swelling by itself and dramatically enhances MPTP in the presence of Ca^++^. On the other hand, AG2 did not induce MPTP by itself but it induces MPTP only in the presence of Ca^++^. We have not attempted to identify the structures of both AG1 and AG2. Depending on the MPTP inducing capacity, AG2 seems a less potent derivative of ATR compared to AG1. According to Ehigie *et al* 18 and Ying *et al* 27, the opening of MPTP is a critical stage of apoptosis that leads to cell death. This suggests that AG1 may produce apoptosis via MPTP opening and mitochondrial swelling which may explain in part AG toxicity in acute poisoning cases.

### Cytotoxicity and genotoxicity test

Cytotoxicity and genotoxicity of AG aqueous rhizome extract was evaluated on *A. cepa* by analyzing four endpoints: root growth and cell morphology at effective concentration (EC50%), percentage of Mitotic Index (MI%), percentage of Mitotic Phases (MP%) and percentage of Total chromosomal and mitotic Aberrations (TA%). As reported by Leme and Marin-Morales 28 and Owolarafe *et al* 29, MI% is a valuable parameter to determine the cytotoxicity of an agent on *A. cepa*. Therefore, the observed dose-dependent decrease in MI% of *A. cepa* roots treated with AG aqueous extract is considered as cytotoxic effect that led to inhibition of roots growth. This indicates cell division cycle delay which is confirmed by the significant decrease of dividing cells percentage compared to negative control and the accumulation of cells in interphase with the three tested concentrations of AG extract. These results confirm those of Sabeen *et al.* 30 where MI decreased significantly in *A. cepa* roots exposed to extracts of selected vegetables. Macroscopic observations of *A. cepa* roots cell morphology revealed brownish roots with dark tips (6 to 12 mg/ml), swollen, friable roots and breaks (10 and 12 mg/ml), that confirm severe cytotoxicity to *A. cepa* roots by AG extract at these concentrations. Since no recovery of root growth was observed after 24 h at concentrations higher than 6 mg/ml, this indicates the cell death of root tips in *A. cepa*. Significant accumulation of cells in interphase and the increased prophase percentage within dividing cells at 6 and 12 mg/ml concentrations may be explained by dysfunction of checkpoints, which may further result in cell death 31. According to Siddiqui *et al* 32 ATP consumption in a dividing cell is much higher than in non-proliferated cell, so ATP shortage may be one of the reasons for MI decrease. As the methanolic extract, the aqueous extract of *A. gummifera* rhizome contains polyphenols: flavonoids, anthocyanins and richer in tannins 5, 21, 22. According to Lefranc *et al.* 33, it also contains polysaccharides, terpenes (ATR and CATR) 24, 33, and essential oils 20, 33. Mitodepressive effect and cell cycle defects observed on A. cepa roots exposed to AG can be attributed to ATR and/or CATR if present; that interact with mitochondrial protein ANT and resulted in ATP exchange inhibition, thereby blocking oxidative phosphorylation, which further prevents the synthesis of ATP and ultimately cell death 32. These results are in agreement with the findings of Park *et al* 33; they reported that ATP depletion interrupt cell cycle, especially during the G1 phase and the G2 to mitosis transition. One of the major cell cycle progression regulators are cyclin-dependent kinases, which phosphorylate substrates and utilize ATP as a donor of phosphoryl group. At low ATP, mitotic arrest leads to mitotic slippage and cells stop growing or extend the duration of the cell cycle until sufficient ATP can be produced. According to Barnum and O'Connell 31, cell death may occur during prolonged duration of the cell cycle.

For genotoxicity assay in the present study, clastogenic, aneugenic and turbagenic changes are evaluated. Chromosome and mitotic aberrations produced by AG on *A. Cepa* roots were significantly higher in the three tested concentrations. The most frequently seen type of chromosomal aberrations was sticky chromosomes, C-mitosis, laggards and micronuclei. Breaks, bridges, vagrant chromosomes, abnormal nuclei, fragmented nucleus, and nuclear buds were also recorded. As discussed elsewhere 34, 35, bridges, stickiness and breaks result of chromatin damage. Bridges result from chromosome and/or chromatid breakage, dicentric chromosome presence or unequal chromatids translocation. Stickiness attributed to the condensation, depolymerization of chromosomal DNA, and entanglement of inter-chromosomic interactions 13. They are irreversible and lead to cell death as highly toxic effect 36. Stickiness can also be caused by chromosomes losing their movement abilities. Inappropriate protein-protein interaction, excessive formation of nucleoproteins and inter-chromosomal linkages of sub-chromatid strands can also cause stickiness 37. C-mitosis, also called stathmokinesis and laggard chromosome are due to spindle failure and increasing risk for aneuploidy 35. High AG dose increases the incidence of lagging chromosomes. Spindle fiber organization and movement are ATP dependent processes. This explains the organization of chromosomes at the metaphase plate and migration of chromosomes during anaphase in AG-treated root tip cells. and chromosome segregation during mitosis will result in lagging chromosome, sticky chromosomes, and bridge formation 32. Breaks indicate clastogenic effect of AG and together with micronucleus (MN) both are clastogenic and aneugenic markers. High frequency of MN indicates that some cells can enter in mitotic phase despite of DNA damage 38. Large multinucleate cells are the result of cell cycle asynchrony, including internuclear asynchrony. Aberrant division of the spindle in early anaphase leads to binucleated cells or the inhibition of cytokinesis after telophase 36. Nuclear buds are markers of polyploidization events and gene amplification, and their formation leads to the expulsion of excess genetic material from aneuploid cells 36, 37. According to the present data, AG induced MPTP opening in bovine mitochondria; furthermore, it induced serious chromosome and mitotic aberrations that are due to ATP shortage as stickiness, bridges, C-mitosis, etc. Swelling in plant mitochondria and proteins liberation is linked to the programmed cell death, this leads us to think about the effect and mechanisms exerted by AG in mammalian mitochondria and in plant mitochondria. Zancani *et al.* reported that swelling of mitochondria occurs in bovine and in some plants (Oat leaves, Etiolated pea stem, Wheat roots and Potato tuber), but in isolated mitochondria permeability transition has been observed in only a few cases and in plants 39. Virolainen et al. also reported that Ca^2+^ increases swelling and cytochrome c release in *Triticum aestivum* L. root mitochondria under anoxic stress and they suggest that the induction of MPTP and cytochrome c liberation can occur via several mechanisms and need specific conditions in both plant and animal tissues 40. These findings led to verify the hypothesis that the mitodepressive effect of AG and its cytotoxic and genotoxic effect are in part due to ATP shortage via MPTP opening.

## Conclusion

In conclusion, this work highlights cellular and mitochondrial adverse effects of *Atractylis gummifera* extracts. Purified AG1 fractions that likely corresponds to ATR derivatives induce MPTP opening leading to swelling of mitochondria and its dysfunction. *Allium cepa* test provides the evidence for *A. gummifera* genotoxicity and cytotoxicity; it is shown that AG have mitodepressive effect on *A. cepa* and it induces several chromosomal aberrations, mitotic cellular abnormalities which suggest its high toxicity. Further studies are required to confirm the link between induction of MPTP and genotoxicity of A. gummifera.
